# Evolution of the B-Block Binding Subunit of TFIIIC That Binds to the Internal Promoter for RNA Polymerase III

**DOI:** 10.1155/2014/609865

**Published:** 2014-02-12

**Authors:** Sachiko Matsutani

**Affiliations:** Division of Microbiology, National Institute of Health Sciences, Tokyo 158-8501, Japan

## Abstract

Eukaryotic RNA polymerase III transcribes tRNA genes, and this requires the transcription factor TFIIIC. Promoters are within genes, with which the B-block binding subunit of TFIIIC associates to initiate transcription. The binding subunits are more than 1000 amino acids in length in various eukaryotic species. There are four regions with conserved sequence similarities in the subunits. The helix-turn-helix motif is included in one of these regions and has been characterized as the *B-block_TFIIIC* family in the Pfam database. In the NCBI and EMBL translated protein databases, there are archaeal proteins (approximately 100 amino acids in length) referred to as B-block binding subunits. Most of them contain a *B-block_TFIIIC* motif. DELTA-BLAST searches using these archaeal proteins as queries showed significant multiple blast hits for many eukaryotic B-block binding subunits on the same proteins. This result suggests that eukaryotic B-block binding subunits were constituted by repeating a small unit of *B-block_TFIIIC* over a long evolutionary period. Bacterial proteins have also been annotated as B-block binding subunits in the databases. Here, some of them were confirmed to have significant similarities to *B-block_TFIIIC*. These results may imply that part of the RNAP III transcription machinery existed in the common ancestry of prokaryotes and eukaryotes.

## 1. Introduction

While bacteria and Archaea have their inherent single RNA polymerases, eukaryotes have multiple types of RNA polymerase. Eukaryotic RNA polymerase III (RNAP III) is one of them and synthesizes tRNA, 5S ribosomal RNA, and other small RNAs (for review [1, 2]). Mammalian short interspersed elements (SINEs), which are retrotransposons, are also transcribed by RANP III [2]. Most RNAP III promoters are inside the sequences expressed as RNAs, and these internal promoters can be divided into three categories based on their organization and transcription factor dependence [3]. In the category of tRNA genes and SINEs, there are internal promoters (type II promoters) consisting of the A- and B-blocks of short nucleotide sequences. A- and B-block sequences are well conserved in various eukaryotes. These promoters are recognized directly by the transcription initiation factor TFIIIC, which is a six-subunit protein [4, 5]. Investigations of TFIIIC assembly on DNA in yeasts and human have demonstrated that B-block binding subunit of TFIIIC first associates with the B-block of the internal promoter (see also Table 1) [1]. TFIIIC bound to DNA recruits another transcription factor TFIIIB, and then TFIIIB assembles RNAP III at the start site of transcription.

**Figure 1:**
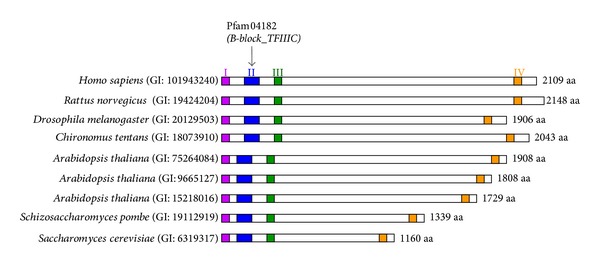
Four regions with conserved sequence similarities in the eukaryotic B-block binding subunits. The N-terminal region (shown as region I in the figure), near the N-terminal region (regions II and III), and near the C-terminal region (region IV) [10]. In the Pfam database, which is a large collection of protein families, the eukaryotic B-block binding subunits are shown to contain the specific HTH motif classified as the *B-block_TFIIIC *family (PF04182) (http://pfam.sanger.ac.uk/family/PF04182#tabview=tab0). The motif is in region II in most eukaryotic subunits. GI: 101943240 and GI: 75264084 are the numbers updated from GI: 4753161 and GI: 25402830, respectively [10].

When the cDNAs for the B-block binding subunits of the yeast, rat, and human TFIIICs were cloned in previous studies, their amino acid sequences were compared for similarities (Table 1) [6–9]. However, these sequences are diverse, and no homology was detected between the yeast and mammalian subunits [7, 8]. Four conserved regions were subsequently identified in the B-block binding subunits of animals, plants, and fungi using the accumulated nucleotide and amino acid sequence data of genomes: three conserved regions are located in the N-terminal one-third regions of the subunits and one is in the C-terminal regions (Figure 1) [10]. However, no DNA binding motifs were detected in any of the four regions. Recently, improved programs for motif detection, such as CD-search, revealed that one of the four regions has a helix-turn-helix (HTH) motif, which forms a typical DNA binding structure (region II in Figure 1). This region is shown as a family of *B-block_TFIIIC* (PF04182) belonging to the clan *HTH* (CL0123) in the Pfam database, which is a large collection of protein families (http://pfam.sanger.ac.uk/): there are currently 324 sequences from 262 species in this family. This domain is considered to directly associate with the B-block sequence of the internal promoter for RNAP III.

RNAP III is generally known to be present in eukaryotes, but not in prokaryotes. However, archaeal and bacterial hypothetical proteins which have been defined or annotated as B-block binding subunits can be found in the translated protein databases of NCBI and EBI. Although some of the information in these databases has not yet been reviewed or confirmed, several proteins have been shown to be members of the *B-block_TFIIIC* family in the Pfam database (http://pfam.sanger.ac.uk/family/PF04182#tabview=tab7). Interestingly, the promoter sequences of the A- and B-blocks are conserved in bacterial tRNA genes, and bacterial tRNA genes can serve as templates for eukaryotic RNAP III [11].

In this study, the amino acid sequences of the eukaryotic B-block binding subunits and their possible prokaryotic relatives were investigated in silico for similarities, and their structural relationships are reported.

## 2. Methods

### 2.1. In Silico Analysis

The programs used to compare the primary structures of proteins were as follows: DELTA-BLAST on the NCBI website at http://blast.ncbi.nlm.nih.gov/Blast.cgi?PROGRAM=blastp&BLAST_PROGRAMS=deltaBlast&PAGE_TYPE=BlastSearch&LINK_LOC=BlastHomeAd [12]; Clustal Omega [13] in the EBI website at http://www.ebi.ac.uk/Tools/msa/clustalo/; and Pfam sequence search in the website at http://pfam.sanger.ac.uk/search?tab=searchProteinBlock [14]. The search set chosen and algorithm parameters used in each of the searches have been described in Section 3. The alignment parameters in Clustal Omega were used at the default settings. CD-search is the NCBI's interface and this is used to search the Conserved Domain Database for protein or nucleotide query sequences (http://www.ncbi.nlm.nih.gov/Structure/cdd/wrpsb.cgi) [15]. DELTA-BLAST performs CD-search to construct a position-specific score matrix (PSSM) and then searches a sequence database using the PSSM [12]. The results of CD-search, which were displayed together with those of DELTA-BLAST, were used in this study. DELTA-BLAST was always performed with one iteration. Neighbor-joining phylogenetic tree was constructed by using the Clustal W program in the DDBJ website of http://clustalw.ddbj.nig.ac.jp/index.php?lang=ja and NJplot [16–18]. The alignment parameters were used at the default settings.

The databases used were the NCBI protein database (http://www.ncbi.nlm.nih.gov/protein) and UniProtKB/TrEMBL (http://www.uniprot.org/).

## 3. Results

### 3.1. B-Block Binding Subunit-Like Proteins in Prokaryotes

As described in Section 1, several archaeal and bacterial proteins are shown to belong to the *B-block_TFIIIC* family in the Pfam database (see http://pfam.sanger.ac.uk/family/PF04182#tabview=tab7). To date, many archaeal sequences have been defined or annotated as B-block binding subunits in the protein database of NCBI.  Table 2 shows the features of representative sequences. CD-search showed that these archaeal proteins significantly matched* B-block_TFIIIC *(Table 2). Similar results were obtained in the Pfam sequence searches (Table 2). These results confirmed that the archaeal proteins examined here were related to the eukaryotic B-block binding subunit at the amino acid sequence level. However, they were between 100 and 200 amino acids (aa) in length, while eukaryotic subunits are more than 1000 aa in length (Table 2; Figure 1).

Bacterial sequences are also defined or annotated as B-block binding subunits in the NCBI protein database (Section 1). However, much of the information on these proteins has not yet been reviewed and CD-search frequently did not hit *B-block_TFIIIC* (data not shown). In the Pfam database of *B-block_TFIIIC*, there are two bacterial proteins (http://pfam.sanger.ac.uk/family/PF04182#tabview=tab7). The representative proteins in which *B-block_TFIIIC* was detected by the CD-search or Pfam sequence search are shown in Table 2. These searches were performed under default conditions. The bacterial proteins were short in length (between 100 and 200 amino acids) (Table 2). *B-block_TFIIIC* was not detected in any protein by both programs, and the *E*-values obtained by these searches were mostly higher than those of archaeal proteins (Table 2). Archaeal proteins appeared to be more similar than bacterial proteins to the eukaryotic *B-block_TFIIIC* sequences. The archaeal and bacterial proteins referred to here were predicted from coding DNA sequences, and therefore, whether these proteins are actually present in cells and have some functions in vivo remains unknown.

### 3.2. Structural Relationship between the Eukaryotic B-Block Binding Subunit and Archaeal Protein

**Figure 2:**
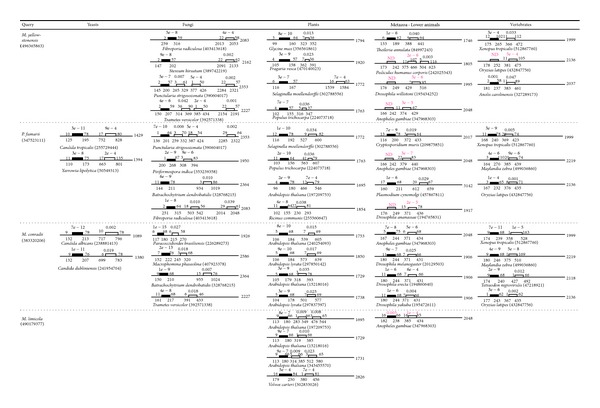
Eukaryotic B-block binding subunits showing similarities in regions different from their authentic *B-block_TFIIIC* sites to archaeal B-block binding subunits. DELTA-BLAST searches were performed in the eukaryotic database (taxid: 2759) using the *M. yellowstonensis*, *P. fumarii*, *M. conradii*, and *M. limicola* sequences shown in Table 2 as queries and the phrase “B-block binding” as an Entrez query. The other conditions were set as the default. All of the displayed blast-hit alignments were examined by eye to identify proteins in which regions different from the authentic *B-block_TFIIIC* sites were detected. Eukaryotic subunits that had such alignments with *E*-values lower than 0.05 are shown. The numbers in parentheses are GI numbers. The horizontal lines represent eukaryotic proteins, and the aa lengths are shown to the right. Small filled boxes represent the alignments between authentic *B-block_TFIIIC* sites. Small empty boxes are alignments in regions different from the authentic *B-block_TFIIIC* sites of eukaryotic proteins. Small grey boxes show authentic *B-block_TFIIIC* sites that were not detected by archaeal proteins. The aa positions of alignments in eukaryotic and archaeal sequences are shown below and above the boxes, respectively. *E*-values of alignments are shown just above the boxes. *E*-values are colored magenta when regions different from the authentic *B-block_TFIIIC* sites were detected at lower *E*-values than the alignments of authentic regions in the same eukaryotic proteins. Because of limited space, the maximum number of proteins shown in each column of the eukaryotic groups against each query is four. More than four proteins were detected in some cases, and fewer or no proteins were detected in other cases. Although the proteins were principally picked up from the displayed alignments in order from the lowest to the higher *E*-values, some disorders were taken for intelligible presentation in the text.

As described above, archaeal B-block binding subunits are approximately 100–200 aa in length, while eukaryotic subunits are more than 1000 aa in length. Region II in the eukaryotic subunit, which is approximately 100 aa in length, contains the *B-block_TFIIIC* motif (Figure 1; Section 1). DELTA-BLAST was used to examine sequence similarities between eukaryotic and archaeal B-block binding subunits. Searches were conducted on the nonredundant protein sequences of eukaryotes (taxid: 2759) using the archaeal proteins shown in Table 2 as queries and the phrase “B-block binding” as an Entrez query. This Entrez query was used to restrict the search to a subset of proteins referred to as “B-block binding” in the database. Other conditions were set as default. Authentic *B-block_TFIIIC* regions in most of organisms, which are annotated in the NCBI protein database, were primarily identified with the lowest *E*-values (data not shown). However, there were several cases in which the archaeal sequence did not hit the *B-block_TFIIIC* regions annotated in the NCBI database but showed significant hits for other regions in the same eukaryotic B-block binding subunits. For example, when the *Metallosphaera yellowstonensis* sequence (GI: 496365863 in Table 2) was used as a query, in the sequences of *Pediculus humanus corporis *(GI: 242025343), *Drosophila willistoni *(GI: 195434252), *Anopheles gambiae* (GI: 347968303), and *Oryzias latipes* (GI: 432847756), the regions of aa positions 375–466, 429–516, 374–429, and 381–475 had significant *E*-values of 2*e* ^−6^, 3*e* ^−6^, 3*e* ^−5^, and 5*e* ^−4^, respectively (Figure 2). On the other hand, the *B-block_TFIIIC *regions of the four proteins, which are annotated in the NCBI protein database (aa positions 173–242, 176–249, 166–242, and 178–252), were not detected (Figure 2). CD-search was performed and confirmed that the annotations of the four proteins are correct (data not shown). These results are shown in Figure 2 and Supplementary Figure  1 (see Supplementary Material available online at http://dx.doi.org/10.1155/2014/609865): a combination of the *Pyrolobus fumarii *sequence (GI: 347523111 in Table 2) and the *Anopheles gambiae* sequences (GI: 347968303) or *Drosophila ananassae *sequence (GI: 194765831); a combination of the *Methanoplanus petrolearius *sequence (GI: 307353829 in Table 2) and the *D. willistoni *sequence (GI: 195434252) or *Nasonia vitripennis* sequence (GI: 345495267); a combination of the *Methanosalsum zhilinae* sequence (GI: 335930125 in Table 2) and the *Drosophila yakuba* sequence (GI: 195472611) or *Drosophila melanogaster* sequence (GI: 20129503); a combination of the *Methanofollis liminatans* sequence (GI: 490137988) and *A. gambiae* sequence (GI: 347968303); a combination of the *Methanoregula formicicum* sequence (GI: 432331009 in Table 2) and the *Pediculus humanus corporis *sequence (GI: 242025343) or *A. gambiae* sequence (GI: 347968303).

As described above, the authentic *B-block_TFIIIC* region of the* A. gambiae* sequence (GI: 347968303) was not detected in DELTA-BLAST searches using *M. yellowstonensis*, *P. fumarii*,* M. liminatans*, and *M. formicicum* sequences as queries. However, in the search using the *Methanocella conradii* sequence (GI: 383320206 in Table 2) as a query, the authentic region in the* A. gambiae* sequence was hit at a significant *E*-value (7*e* ^−8^) (Figure 2). There was another significant hit (*E*-value of 5*e* ^−6^) in this case and the mosquito hit region mostly overlapped with regions that were detected with the *M. yellowstonensis*,* P. fumarii*,* M. liminatans*, and *M. formicicum* sequences (Figure 2; Supplementary Figure 1). Similar results were obtained, for example, with respect to the *D. melanogaster *and *D. yakuba* sequences (GI: 20129503 and GI: 195472611) when DELTA-BLAST searches were performed using the *M*. *zhilinae *and* M. conradii* sequences as queries (Figure 2; Supplementary Figure 1). Not a few eukaryotic B-block binding subunits showed significant blast hits for both of their authentic *B-block_TFIIIC*s regions and one or more regions different from the authentic region in the same sequences (Figure 2; Supplementary Figure 1): for example, the *M. yellowstonensis* sequence hit the fungal *Punctularia strigosozonata* sequence (GI: 390604017) in four regions, and the *P. fumarii* sequence hit the yeast *Candida tropicalis *sequence (GI: 255729444) in two regions.

**Figure 3:**
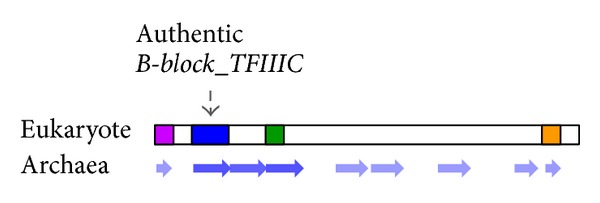
Highly schematized cartoon showing distribution of the archaeal B-block binding subunit sequence in the eukaryotic subunit. Colored regions in the eukaryotic subunit correspond to those in Figure 1. Based on all of the results shown in Figure 2 and Supplementary Figure  1, the archaeal subunits (shown as arrows) are placed below the eukaryotic subunit. Arrows are colored more intensely where BLAST hits were frequently detected with lower *E*-values.

These results showed that many B-block binding subunits from yeasts to vertebrates have one or more *B-block_TFIIIC-*like regions in addition to the authentic *B-block_TFIIIC* regions in their entire sequences. Although the *B-block_TFIIIC*-like regions were repeated near the authentic *B-block_TFIIIC *regions in many cases, they also existed in the C-terminal regions and in the middle regions of the subunits (Figure 3).

### 3.3. Comparison of the Primary Structures of Archaeal B-Block Binding Subunits with Those of the Regions Conserved in Eukaryotic B-Block Binding Subunits

**Figure 4:**
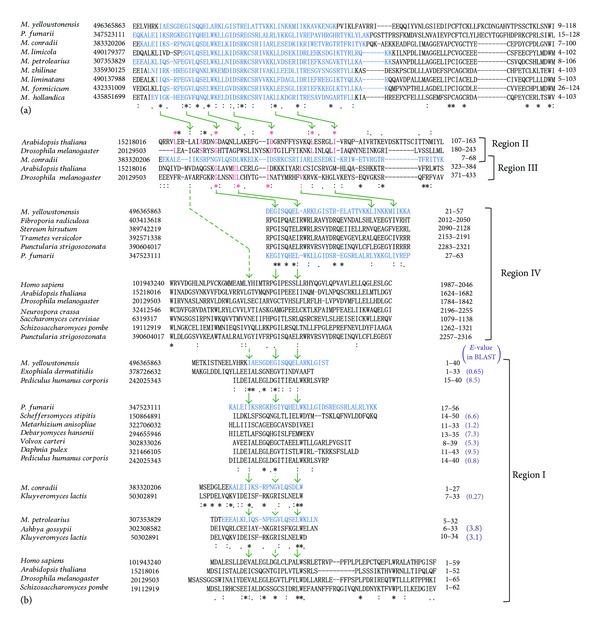
Comparison of the primary structures of the archaeal B-block binding proteins with the regions conserved in the eukaryotic B-block binding subunits. Each of the scientific names is followed by the GI number, aa sequence, and aa positions. The *B-block_TFIIIC* regions in the archaeal proteins are colored in blue (see Table 2). Green arrows indicate common residues both in Archaea and eukaryotic conserved regions. Green dotted arrows mean no conservation between the two alignments just above and just below the arrows. (a) Clustal Omega alignment of archaeal proteins defined or annotated as B-block binding subunits in the NCBI protein database. Amino acid residues of the N-terminal and C-terminal ends which were not conserved in the nine protein sequences were cropped in this alignment. (b) Clustal Omega alignments of the archaeal sequences with each of regions I, II, III, and IV in the eukaryotic B-block binding subunits. Regions II and III of the *A. thaliana* and *D. melanogaster* proteins (GI: 15218016 and GI: 20129503) were used for comparison with archaeal proteins. This is because these sequences are used in the previous alignments of regions II and III (Figure 3 in [10]) and were detected in DELTA-BLAST searches using the *M. conradii *sequence as a query in this study (Figure 2). These two proteins could precisely link the previous regions II and III alignments to the present alignment with the archaeal sequence. The alignments of regions II and III are shown in a combined form via the *M. conradii* sequence for clarity. Amino acid residues shown in magenta were conserved also in the alignments of Figures  3B and 3C in [10]. For comparison of the archaeal sequences with region IV, the C-terminal regions of the fungal proteins which were detected at significant *E*-values in Figure 2 were aligned with their related archaeal sequences. Just below the alignment, the C-terminal regions of several eukaryotic B-block binding subunits (region IV) were aligned (see also Figure 3D in [10]). To examine whether the archaeal proteins are related to region I, the N-terminal regions of the eukaryotic B-block binding subunits which were simultaneously detected with the authentic *B-block_TFIIIC* regions were visually searched for from the results of DELTA-BLAST. Clustal Omega was then performed. *E*-values of the matches to the N-terminal regions in DELTA-BLAST were higher than threshold (shown in parentheses next to the aa positions of the Clustal alignments). An alignment of the N-terminal regions of several eukaryotic B-block binding subunits (region I) is shown at the bottom of the Figure (see also Figure 3A in [10]).

The archaeal B-block binding subunits shown in Table 2 commonly contain a *B-block_TFIIIC* motif which is mainly in the N-terminal halves of the sequences (Figure 4(a)). In DELTA-BLAST searches in Section 3.2 these motif regions always hit the eukaryotic subunit sequences (Figure 2; Supplementary Figure 1). The amino acid residues conserved in these archaeal sequences (see Figure 4(a)) corresponded well to those conserved in the *B-block_TFIIIC* family (see http://pfam.sanger.ac.uk/family/PF04182#tabview=tab3). As described in Section 1, the eukaryotic B-block binding subunits contain four regions with conserved sequence similarities [10], with one of these regions (region II) being the authentic *B-block_TFIIIC* (Figure 1). However, the archaeal sequences frequently hit regions different from the authentic *B-block_TFIIIC* sites in the eukaryotic subunits (Section 3.2). Therefore, it was examined whether the other conserved regions (regions I, III, and IV in Figure 1) have sequence similarities to the archaeal proteins.

The *Arabidopsis thaliana* sequence of GI: 15218016 and *D. melanogaster* sequence of GI: 20129503 were previously used in Clustal W alignments of regions II and III [10]. In this study, these two sequences were hit with DELTA-BLAST using the *M. conradii* sequence (GI: 383320206) as a query: two regions were detected in each of these proteins (*A. thaliana* aa positions 105–179 and 318–393 and *D. melanogaster* aa positions 180–244 and 371–431) (Figure 2). When the amino acid sequences of the detected regions were searched in the previous alignments shown in Figure 3 of Matsutani [10] by eye, the former sequence corresponded to regions II and the latter corresponded to region III in each of the proteins. The combined alignment of regions II and III in the *A. thaliana* and *D. melanogaster* sequences via the *M*. *conradii* sequence is shown at the top of Figure 4(b): the amino acid residues that are conserved well in the *B-block_TFIIIC *family (http://pfam.sanger.ac.uk/family/PF04182#tabview=tab3) were conserved in this alignment. They were also conserved in regions II and III alignments of Figures 3B and 3C in [10]. These results suggest that the *B-block_TFIIIC-*like sequence commonly exists as region III in eukaryotic B-block binding subunits (Figure 3).

The C-terminal regions of several fungal B-block binding subunits were hit with *E*-values lower than threshold in DELTA-BLAST searches using the archaeal sequences as queries (Figure 2): when the *M*. *yellowstonensis* sequence (GI: 496365863) was used as a query, in the sequences of *Fibroporia radiculosa* (GI: 403413618, 2083 aa), *Stereum hirsutum *(GI: 389742219, 2162 aa), *Punctularia strigosozonata* (GI: 390604017, 2353 aa), and *Trametes versicolor* (GI: 392571338, 2227 aa), the regions of aa positions 2013–2053, 2091–2133, 2284–2321, and 2154–2191 had significant *E*-values of 4*e* ^−4^, 0.002, 0.002, and 0.001, respectively. When the *P. fumarii* sequence (GI: 347523111) was used as a query, the C-terminal region (aa positions 2285–2322) of the *P. strigosozonata* sequence (GI: 390604017, 2353 aa) was hit at an *E*-value of 0.002 (Figure 2). These fungal C-terminal regions were aligned with the *M. yellowstonensis* and *P. fumarii *sequences using Clustal Omega. The alignments are shown in the middle of Figure 4(b): the amino acid residues conserved in the archaeal sequences and regions II and III were conserved in these fungal C-terminal regions, which corresponded to the latter half of region IV alignment in Figure 3C of [10].

There were no hits in the N-terminal regions of the eukaryotic B-block binding subunits at *E*-values lower than threshold in DELTA-BLAST searches using the archaeal sequences as queries. The N-terminal regions in several subunits, were hit at *E*-values higher than threshold together with the authentic *B-block_TFIIIC* regions showing significant *E*-values. For example, aa positions 1–36 of the *M. yellowstonensis* sequence (GI: 496365863) were similar to aa positions 1–33 of the *Exophiala dermatitidis *sequence (GI: 378726632) (*E*-value of 0.65), and aa positions 1–27 of the *M. conradii* sequence (GI: 383320206) were similar to aa positions 7–33 of the *Kluyveromyces lactis *sequence (GI: 50302891) (*E*-value of 0.27). All such N-terminal regions are shown in the lower part of Figure 4(b) as alignments with their relevant archaeal sequences. The amino acid residues conserved in the archaeal sequences and regions II, III, and IV in the eukaryotic B-block binding subunits were conserved in these alignments, although their lengths were short (Figure 4(b)). Furthermore, these alignments were compared with the region I alignment shown in Figure 3A of [10]. These alignments appeared to correspond to the former half of the region I alignment (see the bottom of Figure 4(b)).

Sequences similar to the archaeal B-block binding subunits were sometimes detected out of the four conserved regions in the eukaryotic B-block binding subunits (Figure 2; Supplementary Figure  1): for example, aa positions 16–63 of the *M. yellowstonensis* sequence (GI: 496365863) were similar to aa positions 1539–1584 of the *Selaginella moellendorffii *sequence (GI: 302788556, 1772 aa) (*E*-value of 7*e* ^−4^), and aa positions 3–86 of the *P. fumarii *sequence (GI: 347523111) were similar to aa positions 934–1019 of the *Batrachochytrium dendrobatidis *sequence (GI: 328768215, 2346 aa) (*E*-value of 0.010). All these results suggest that the eukaryotic B-block binding subunit was mainly constructed by repeated duplication of the *B-block_TFIIIC *sequence (Figure 3). 

### 3.4. Investigation of the Primary Structures of the Bacterial B-Block Binding Subunits

**Figure 5:**
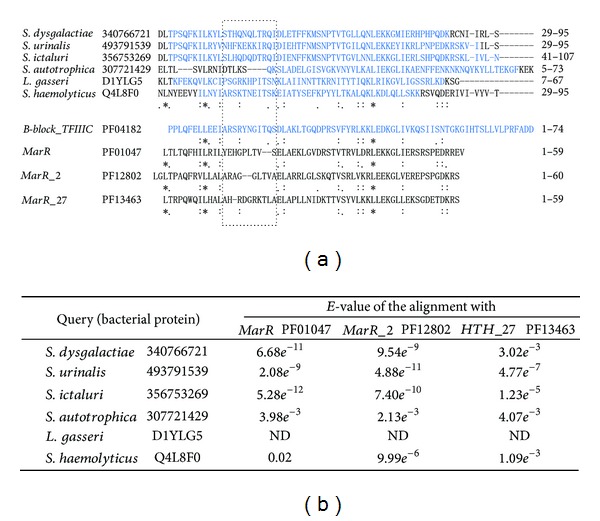
Comparison of the primary structures of the bacterial B-block binding proteins with the *B-block_TFIIIC* motif and other HTH motifs. (a) Clustal Omega alignment of the six bacterial proteins provided in Table 2. The relevant region of the alignment is only shown. Amino acid residues conserved in the alignment are indicated by asterisks, colons, and dots. Clustal Omega was performed also using the six bacterial proteins and each cdd sequence of the *B-block_TFIIIC *(PF04182), *MarR *(PF01047), *MarR_2* (PF12802), and *HTH_27* (PF13463) families. The alignments were reviewed by eye, and motif sequences with marks showing amino acid conservation were placed beneath the alignment constructed first from the bacterial proteins only. The cdd sequences were obtained from the CD-search results. Each of the bacterial names is followed by the GI number (or entry name), aa sequence, and aa positions. HTH motifs are also similarly represented. The *B-block_TFIIIC* regions are colored in blue (see Table 2). (b) *E*-values of the alignments of bacterial B-block binding proteins with *MarR*, *MarR_2*, or *HTH_27* in CD-search.

As already described and shown in Table 2, the NCBI and EBI protein databases include bacterial proteins defined or annotated as B-block binding subunits. Similarities between the bacterial proteins shown in Table 2 and the *B-block TFIIIC* motif seemed to be unclear, because the *E*-values were mostly higher than those of archaeal subunits to the *B-block TFIIIC *motif, and both of the CD-search and Pfam search did not detect a *B-block TFIIIC *motif for each of the queries (Table 2). Therefore, possible similarities were investigated using Clustal Omega. The six bacterial B-block binding subunits in Table 2 were first aligned with themselves. The six bacterial proteins were then aligned with the *B-block_TFIIIC* cdd sequence (conserved domain's consensus sequence) which was shown in CD-search. As shown in Figure 5(a), the amino acid residues conserved in the alignment consisting only of bacterial proteins corresponded well to those conserved in the alignment of the bacterial proteins and the *B-block_TFIIIC* sequence. CD-search of the bacterial proteins showed that they have similarities also to other HTH motifs such as *MarR*, *MarR_2*, and *HTH27*, and the *E*-values were frequently lower than those of *B-block_TFIIIC* (Table 2 and Figure 5(b)). Like *B-block_TFIIIC*, *MarR* (PF01047), *MarR_2* (PF12802), and *HTH_27* (PF13463) are members of the clan *HTH *(CL0123) (http://pfam.sanger.ac.uk/clan/CL0123#tabview=tab0). Proteins with the *MarR* and *MarR_2* motifs are involved in resistance to multiple antibiotics. They repress the expression of *mar* operons consisting of antibiotic-resistant genes. *HTH_27* is a family of the winged helix-turn-helix motif. Each of the cdd sequences of* MarR*, *MarR_2*, and *HTH_27* obtained as representatives from CD-search was aligned with the six bacterial B-block binding subunits (Figure 5(a)). The amino acid residues conserved in the alignment consisting only of bacterial proteins corresponded to those conserved in the alignments of the bacterial proteins and the *MarR*, *MarR_2*, and *HTH_27 *sequences, except in the left central regions of the alignments (Figure 5(a)). As shown in the boxed region in Figure 5(a), the two columns with amino acid preferences were common in both of the alignment consisting only of the bacterial proteins and that constructed with the bacterial proteins plus *B-block_TFIIIC*. These amino acid preferences are shown in the *B-block_TFIIIC* family (see http://pfam.sanger.ac.uk/family/PF04182#tabview=tab3). On the other hand, comparably low amino acid preferences are found in the left central regions of the *MarR*, *MarR_2*, and *HTH_27* families (http://pfam.sanger.ac.uk/family/PF01047#tabview=tab4, http://pfam.sanger.ac.uk/family/PF12802#tabview=tab4, and http://pfam.sanger.ac.uk/family/PF13463#tabview=tab4). Actually, the corresponding region in the alignment of the bacterial proteins and the cdd sequence of *MarR*, *MarR_2*, or *HTH_27 *did not show such similarities (boxed region in Figure 5(a)). Note that the 15th R residue of the cdd sequence of *HTH_27* is not conserved in the motif (http://pfam.sanger.ac.uk/family/PF13463#tabview=tab4). These results confirmed that the bacterial proteins shown in Table 2 are related to the eukaryotic B-block binding subunit.

## 4. Discussion

Here, the *B-block_TFIIIC* motif sequences of several archaeal short proteins were shown to be repeated in the longer B-block binding subunits of various eukaryotes. This finding suggests that the eukaryotic B-block binding subunit has been constructed by repeating duplication of the *B-block_TFIIIC *region in long evolutionary time. Repetition of *B-block_TFIIIC *sequence was common in various eukaryotic B-block binding subunits. Therefore, the *B-block_TFIIIC* sequence had possibly begun to be repeated in the first primitive eukaryotes. Alternatively, the sequence might have been originally repeated in the ancient B-block binding subunit. It may be imagined that the repetition was lost in the evolutionary process from the ancient organisms to prokaryotes to leave single copies on the subunits. However, this is opposite to the established view that DNA duplications have contributed to the evolution of organisms [19, 20].

Archaea, eubacteria, and eukaryotes have been thought to possess the transcription machinery specific to each of them [21]. Although only one RNA polymerase in Archaea corresponds to the eukaryotic RNAP II, orthologs of the eukaryotic RNAP III subunit Rpc34 are present there [22]. Rpc34, which is a specific and essential subunit of RNAP III, interacts with the transcription factor TFIIIB to participate in RNAP III recruitment [23]. It is suggested that the functional separation of RNAP predates the origin of eukaryotes [22]. Rpc34 contains two domains, which are the N-terminal HTH and the C-terminal Zn-finger domains [22]. Interestingly, HTH regions in several of the archaeal Rpc34s showed the significant similarity to the *B-block_TFIIIC* motif in CD-searches (data not shown). Additionally, the *M. yellowstonensis* sequence (GI: 496365863) used in this study showed similarity to Rpc34: when CD-search was performed, its HTH region was aligned with the HTH region of the Rpc34 motif (PF05158) at an *E*-value of 0.03 (data not shown), although the value was much higher than that of the alignment with *B-block_TFIIIC *(Table 2).

**Figure 6:**
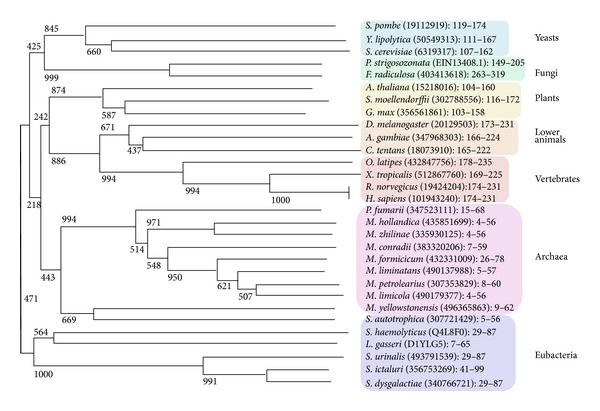
Phylogenetic tree for the alignment of *B-block_TFIIIC* family. The neighbor-joining method was used and bootstrap values are shown in the tree [16–18]. The aa positions of sequences used for Clustal W are indicated next to the subunit GI numbers.

Molecular phylogenetic studies using small subunit rRNA and the proteins like actin and *α*-tubulin place fungi as more closely related to animals than either is to plants [24, 25]. However, it is reported that with respect to the B-block binding subunits of TFIIICs, animals appear to be evolutionarily closer to plants than to fungi [10]. This was shown by the results of PSI-BLAST searches using B-block binding subunits as queries. The Pfam website provides a phylogenetic tree of the family of *B-block_TFIIIC*, also where animals are more closely related to plants than to fungi (http://pfam.sanger.ac.uk/family/PF04182#tabview=tab5). In this tree, prokaryotic *B-block_TFIIIC* sequences are not contained. Therefore, the phylogenetic tree was constructed together with the prokaryotic *B-block_TFIIIC* sequences. The tree also indicated that animals are evolutionarily closer to plants than to fungi (Figure 6). Although Archaea was placed as more closely related to animals and plants than to fungi (Figure 6), this result seems to be less reliable. When DELTA-BLAST searches were performed in the eukaryotic protein database using archaeal B-block binding subunit sequences as queries and the phrase “B-block binding” as an Entrez query, many fungal B-block binding subunits were hit with lower *E*-values than those of animals and plants (data not shown).

Some bacterial proteins in databases were confirmed to contain a *B-block_TFIIIC *motif in this study. The host species belong only to four genera although whole genome sequencing has been completed in many bacterial genera. Why is the B-block binding subunit absent in the other bacteria? Three possibilities are there. The first one is that in the other bacteria genes encoding the B-block binding subunit proteins had been lost. The protein might have been nonessential for bacterial survival. The second possibility is that the B-block binding subunit had highly diverged in the other bacteria, and those protein sequences cannot be detected with the similarity search programs that are currently used. This possibility is extensively discussed in the following paragraph. The third possibility is that horizontal gene transfers between bacteria and Archaea or eukaryotes. A few cases of horizontal gene transfer from Archaea to bacteria have been reported [26]. However, this possibility seems less plausible than the other two possibilities because A- and B-block sequences of the internal promoter are generally conserved in bacterial tRNA genes.

Bacterial IS1 is a mobile DNA (for review, [27]) and appears to possess the RNAP III promoter sequence in the internal region, like bacterial tRNA genes [28]. The RNAP III promoter-like sequence in IS1 acts as a cis-element to stimulate RNA synthesis from promoters located upstream of the cis-element [28, 29]. The RNAP III promoter sequence of Alu, which is a human SINE, also stimulates RNA synthesis in *E. coli *[28]. The product of the *E. coli artA* gene is shown to bind to the internal region of IS1 and stimulate transcription [29, 30]. Although the primary structure of the bacterial ArtA protein was compared with those of the prokaryotic B-block binding subunits in this study, clear similarities were not found. More improved programs to analyze protein structure may clarify these points in the future. When the structures of the eukaryotic B-block binding subunits were previously investigated in silico, the HTH motif was not detected in any program [10].

The relatives of the RNAP III transcription machinery may have existed in the common ancestry of eukaryotes and prokaryotes due to the presence of the *B-block_TFIIIC* motif in archaeal and bacterial proteins and type II promoter sequences in prokaryotic genomes.

## Supplementary Material

Supplementary Figure: Eukaryotic B-block binding subunits showing similarities in regions different from their authentic *B-block_TFIIIC* sites to the B-block binding subunits of the *M. petrolearius, M. zhilinae, M. liminatans, M. formicicum*, and *M. hollandica*. Details are as described in the legend to Figure 2, except that the maximum number of proteins shown in each column of the eukaryotic groups against each query is three.

## Figures and Tables

**Table 1:** B-block binding subunits of TFIIICs in yeasts and vertebrates.

Subunit name (other name)	Organism	Length (aa)	GI number	Reference
TFC3p (*τ*138)	*Saccharomyces cerevisiae *	1160	6319317	[6]
Sfc3p	*Schizosaccharomyces pombe *	1339	19112919	[9]
hTFIIIC220 (TFIIIC*α*, GTF3C1)	*Homo sapiens *	2109	101943240	[8]
rTFIIIC220	*Rattus norvegicus *	2148	19424204	[7]

**Table 2:** Prokaryotic proteins annotated as B-block binding subunits of TFIIICs in the NCBI protein database and UniProtKB/TrEMBL.

Protein name	Source organism	Length (aa)	GI number or entry name	Alignment with *B-block_TFIIIC* ^a^	
				aa positions (query/Pfam04182)	*E*-value
Archaea					
B-block binding subunit of TFIIIC	*Metallosphaera yellowstonensis *	123	496365863	16–63/11–58	2.87*e* ^−4^ (3.2*e* ^−7^)
B-block binding subunit of TFIIIC	*Pyrolobus fumarii* 1A	138	347523111	12–78/1–70	7.93*e* ^−8^ (7.5*e* ^−9^)
B-block binding subunit of TFIIIC	*Methanocella conradii* HZ254	169	383320206	9–68/6–69	7.18*e* ^−7^ (3.3*e* ^−6^)
AsnC family transcriptional regulator	*Methanoplanus limicola *	115	490179377	16–66/17–70	1.11*e* ^−3^ (1.5*e* ^−5^)
Putative AsnC family transcriptional regulator	*Methanoplanus petrolearius* DSM 11571	117	307353829	5–70/1–70	1.07*e* ^−4^ (4.3*e* ^−5^)
Conserved hypothetical protein	*Methanosalsum zhilinae* DSM 4017	115	335930125	8–62/8–66	1.31*e* ^−4^ (4.2*e* ^−6^)
AsnC family transcriptional regulator	*Methanofollis liminatans *	113	490137988	11–66/11–69	1.37*e* ^−7^ (1.7*e* ^−8^)
B-block binding subunit of TFIIIC	*Methanoregula formicicum* SMSP	135	432331009	34–87/13–69	7.32*e* ^−9^ (2.7*e* ^−9^)
B-block binding subunit of TFIIIC	*Methanomethylovorans hollandica* DSM15978	120	435851699	9–62/9–66	3.70*e* ^−5^ (3.3*e* ^−5^)
Bacteria					
B-block binding subunit of TFIIIC	*Streptococcus dysgalactiae *subsp.* equisimilis* SK1250	144	340766721	31–87/1–57	0.02 (ND)
B-block binding subunit of TFIIIC	*Streptococcus urinalis *	151	493791539	31–92/1–62	7.25*e* ^−3^ (ND)
B-block binding subunit of TFIIIC	*Streptococcus ictaluri* 707-05	160	356753269	43–107/1–69	3.40*e* ^−5^ (ND)
AsnC family transcriptional regulator	*Sulfurimonas autotrophica* DSM 16294	102	307721429	20–70/21–71	2.71*e* ^−3^ (ND)
Putative uncharacterized protein	*Lactobacillus gasseri* 224-1	120	D1YLG5	(10–64/2–55)	ND (0.00023)
SarR protein	*Staphylococcus haemolyticus* JCSC1435	114	Q4L8F0	37–81/7–51	0.04 (ND)

^a^Data obtained from the CD-search using the prokaryotic protein sequences as queries. The *E*-values shown in parentheses are data from the Pfam sequence searches. “ND” means not detected. The aa positions of alignments obtained in the Pfam sequence searches are not shown except the case of D1YLG5. Both searches were performed under the default conditions.
