# Supplementary material for: Evolution of the B-Block Binding Subunit of TFIIIC That Binds to the Internal Promoter for RNA Polymerase III
Source: Int J Evol Biol. 2014 Feb 12;2014:609865. doi: 10.1155/2014/609865 (PMC3945056; doi:10.1155/2014/609865)
Supplement: Supplementary file 1 — Supplementary Figure: Eukaryotic B-block binding subunits showing similarities in regions different from their authentic B-block_TFIIIC sites to the B-block binding subunits of the M. petrolearius, M. zhilinae, M. liminatans, M. formicicum, and M. hollandica. Details are as described in the legend to Figure 2, except that the maximum number of proteins shown in each column of the eukaryotic groups against each query is three. [file 609865.f1.pdf]

Supplementary Material

Supplementary Figure: Eukaryotic B-block binding subunits showing similarities in regions different from their authentic *B-block_TFIIIC* sites to the B-block binding subunits of the *M. petrolearius*, *M. zhilinae*, *M. liminatans*, *M. formicicum*, and *M. hollandica*. Details are as described in the legend to Figure 2, except that the maximum number of proteins shown in each column of the eukaryotic groups against each query is three.

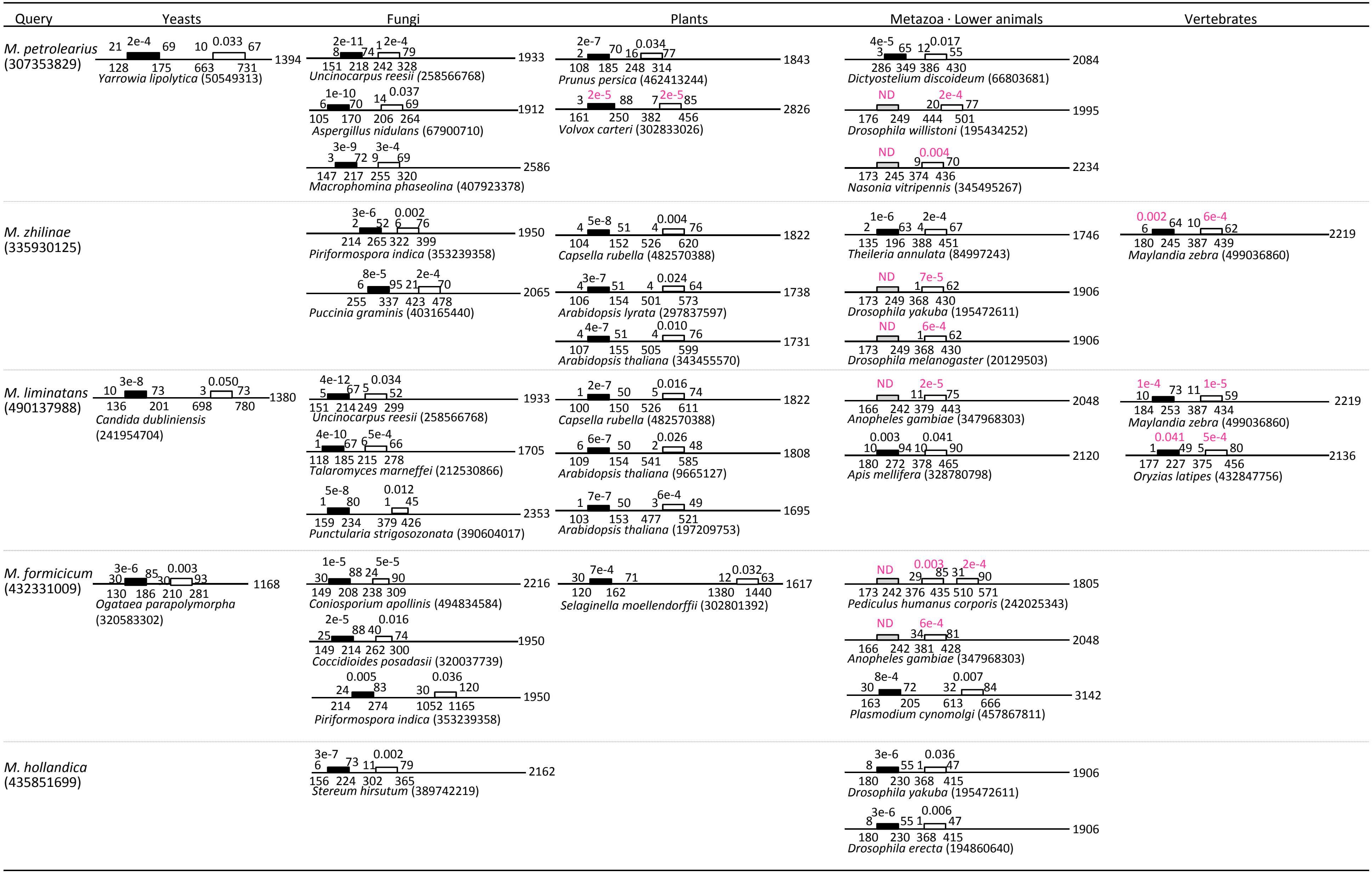
Query
Yeasts
Fungi
Plants
Metazoa · Lower animals
Vertebrates
M. petrolearius (307353829)
Yarrowia lipolytica (50549313)
Uncinocarpus reesii (258566768)
Aspergillus nidulans (67900710)
Macrophomina phaseolina (407923378)
Prunus persica (462413244)
Volvox carteri (302833026)
Dictyostelium discoideum (66803681)
Drosophila willistoni (195434252)
Nasonia vitripennis (345495267)
M. zhilinae (335930125)
Piriformospora indica (353239358)
Puccinia graminis (403165440)
Capsella rubella (482570388)
Arabidopsis lyrata (297837597)
Arabidopsis thaliana (343455570)
Theileria annulata (84997243)
Drosophila yakuba (195472611)
Drosophila melanogaster (20129503)
Maylandia zebra (499036860)
M. liminatans (490137988)
Candida dubliniensis (241954704)
Uncinocarpus reesii (258566768)
Talaromyces marneffei (212530866)
Punctularia strigosozonata (390604017)
Capsella rubella (482570388)
Arabidopsis thaliana (9665127)
Arabidopsis thaliana (197209753)
Anopheles gambiae (347968303)
Apis mellifera (328780798)
Maylandia zebra (499036860)
Oryzias latipes (432847756)
M. formicicum (432331009)
Ogataea parapolymorpha (320583302)
Coniosporium apollinis (494834584)
Coccidioides posadasii (320037739)
Piriformospora indica (353239358)
Selaginella moellendorffii (302801392)
Pediculus humanus corporis (242025343)
Anopheles gambiae (347968303)
Plasmodium cynomolgi (457867811)
M. hollandica (435851699)
Stereum hirsutum (389742219)
Drosophila yakuba (195472611)
Drosophila erecta (194860640)
